# Psychosocial and obstetric determinants of women signalling distress during Edinburgh Postnatal Depression Scale (EPDS) screening in Sydney, Australia

**DOI:** 10.1186/s12884-019-2565-3

**Published:** 2019-11-07

**Authors:** Sarah Khanlari, John Eastwood, Bryanne Barnett, Sabrina Naz, Felix Akpojene Ogbo

**Affiliations:** 1 Department of Community Paediatrics, Sydney Local Health District, Croydon Community Health Centre, 24 Liverpool Street, Croydon, NSW 2132 Australia; 2 School of Medicine and Public Health, University of Newcastle, Callaghan Campus, University Drive, Callaghan, NSW 2308 Australia; 3 Sydney Institute for Women Children and their Families, Sydney Local Health District, 18 Marsden Street, Level 1, Camperdown, NSW 2050 Australia; 4 Ingham Institute for Applied Medical Research, 1 Campbell Street, Liverpool, NSW 2170 Australia; 5 School of Women’s and Children’s Health, Faculty of Medicine, The University of New South Wales, Kensington, NSW 2052 Australia; 6 Menzies Centre for Health Policy, Charles Perkins Centre, School of Public Health, Sydney University, Sydney, NSW 2006 Australia; 7 School of Psychiatry, Faculty of Medicine, University of New South Wales, Kensington, NSW 2052 Australia; 8 Translational Health Research Institute, School of Medicine, Western Sydney University, Campbelltown Campus, Locked Bag 1797, Penrith, NSW 2571 Australia

**Keywords:** Perinatal, Pregnancy, Distress, Depression, EPDS, Screening, Determinants, Psychosocial, Prevention

## Abstract

**Background and objectives:**

The perinatal period presents a high-risk time for development of mood disorders. Australia-wide universal perinatal care, including depression screening, make this stage amenable to population-level preventative approaches. In a large cohort of women receiving public perinatal care in Sydney, Australia, we examined: (1) the psychosocial and obstetric determinants of women who signal distress on EPDS screening (scoring 10–12) compared with women with probable depression (scoring 13 or more on EPDS screening); and (2) the predictive ability of identifying women experiencing distress during pregnancy in classifying women at higher risk of probable postnatal depression.

**Methods:**

We analysed routinely collected perinatal data from all live-births within public health facilities from two health districts in Sydney, Australia (*N* = 53,032). Perinatal distress was measured using the EPDS (scores of 10–12) and probable perinatal depression was measured using the EPDS (scores of 13 or more). Logistic regression models that adjusted for confounding variables were used to investigate a range of psychosocial and obstetric determinants and perinatal distress and depression.

**Results:**

Eight percent of this cohort experienced antenatal distress and about 5 % experienced postnatal distress. Approximately 6 % experienced probable antenatal depression and 3 % experienced probable postnatal depression. Being from a culturally and linguistically diverse background (AOR = 2.0, 95% CI 1.8–2.3, *P* < 0.001), a lack of partner support (AOR = 2.9, 95% CI 2.3–3.7) and a maternal history of childhood abuse (AOR = 1.9, 95% CI 1.6–2.3) were associated with antenatal distress. These associations were similar in women with probable antenatal depression. Women who scored 10 to12 on antenatal EPDS assessment had a 4.5 times higher odds (95% CI 3.4–5.9, *P* < 0.001) of experiencing probable postnatal depression compared with women scoring 9 or less.

**Conclusion:**

Antenatal distress is more common than antenatal depressive symptoms and postnatal distress or depression. Antenatal maternal distress was associated with probable postnatal depression. Scale properties of the EPDS allows risk-stratification of women in the antenatal period, and earlier intervention with preventively focused programs. Prevention of postnatal depression could address a growing burden of illness and long-term complications for mothers and their infants.

## Background

Anxiety and depression are the most common complications of the perinatal period, affecting approximately 10–15% of women [1–3], with a global trajectory of increasing burden [4]. This pooled estimate varies widely between and within countries [5]. When undetected these disorders pose a range of risks to the mother’s health, psychosocial wellbeing, relationships and her infant’s development [6]. In Australia, death by suicide or trauma remain the two leading causes of death in the first postpartum year [7], with the great majority of cases having had psychiatric diagnoses including substance use identified [8]. An increasing body of literature suggests perinatal depression and anxiety interrupt the mother-infant relationship leading to a range of neurocognitive, psychiatric and developmental problems in the offspring of affected mothers [9–12]. Maternal distress has also been shown to contribute to the amplification of parenting stress, birth complications such as premature labour [13] and low birth weight [14], and lead to adverse consequences for child growth and development [15].

Local and international research has confirmed that antenatal depression is more prevalent than postnatal depression peaking in its burden in the third trimester [16–18], suggesting a proportion of women with postnatal depression are experiencing a continuation of symptoms from the antenatal period. The reported prevalence of antenatal and postnatal depression in Australia is 6–17% and 3–11% respectively [17, 19], increasing markedly in culturally and linguistically diverse (CALD) communities. Antenatal depression has been found to be associated with low self-esteem, low levels of social support, negative cognitive style, recent major life events, low income and a maternal history of abuse [20]. Postnatal depression is most strongly predicted by presence of antenatal depression [2, 17, 19–21], and goes on to be the major predictor of parenting stress [20]. While there is now sufficient evidence to reliably identify antenatal depression as a major risk factor for postnatal depression, an area that has been poorly investigated is whether antenatal distress is a risk factor for postnatal depression, and whether there are similarities in the socio-demographic and obstetric determinants of women with perinatal distress compared with perinatal depression. The degree to which co-morbid anxiety contributes to perinatal mood disorder has gained more appropriate attention recently [22]. Distress that is sub-threshold for a diagnosis of major depression may cause significant functional disability as it is more commonly associated with anxiety symptoms [23]. The concept of maternal distress has been described in detail elsewhere [24].

The Edinburgh Postnatal Depression Scale (EPDS) is a self-reported screening questionnaire initially designed for use in the postnatal period [25]. As the tool became more widely available, it demonstrated its utility as a cross-culturally valid tool for use in multiple settings. The Australian National Perinatal Depression Initiative [26] endorsed the use of the EPDS as part of a universally-delivered psychosocial assessment for women receiving maternity care in the public health care system. In these settings, a score of 13 or more merits possible referral for specialised assessment or at least re-application of the EPDS within two to 3 weeks [27]. Validation studies of the EPDS in the antenatal period have justified higher cut-offs due to concern that transient heightened distress in pregnancy will be captured and misclassified [28]. However, heightened anxiety during pregnancy has been linked with poor perinatal outcomes [13, 29, 30] and contributes significantly to the symptom burden [31].

The present study aims to investigate the biopsychosocial and obstetric determinants of antenatal and postnatal distress (defined as EPDS 10 to 12) and probable depression (EPDS 13 or more) in a large and culturally-diverse cohort in Australia. We examine the capacity of an antenatal EPDS score of 10 to 12 predicting development of postnatal depression, defined as an EPDS score of 13 or more.

## Methods

### Data source

This study was based on a retrospective cohort of mothers of all live births (*N* = 53,032) in public health facilities in South Western Sydney Local Health District (SWSLHD) and the Sydney Local Health District (SLHD) in 2014 to 2016, in New South Wales (NSW), Australia. Antenatal data routinely collected by midwives were linked to postnatal data routinely collected by child and family health nurses, using unique individual identifiers that allowed linkage of maternal and child health data from the various services (e.g., antenatal, hospital, postnatal, child and family) attended. The unique individual identifier is a code that does not include personal identification information about either the mother and/or the infant. Antenatal information (such as sociodemographic characteristics, alcohol use, partner support, antenatal health problems, maternal history of childhood abuse, and history of intimate partner violence) were collected by qualified midwives in the first antenatal visit. Similarly, the mode of delivery data was recorded immediately post-birth, while postnatal information (e.g., postnatal depression using the EPDS) was collected by qualified child and family health nurses. These data are stored in the Information Management & Technology Division (IM&TD) database in each health district. After ethics approvals were obtained, the data were cleaned and coded for analysis.

### Study setting

SWSLHD and SLHD represents approximately 52% of the Sydney metropolitan population, with over 1,621,000 residents. This region of Sydney is one of Australia’s most culturally diverse, with approximately half of all mothers born overseas [32, 33]. Mothers from the Middle East & Africa (11%), South East Asia (10%), Southern Asian (8%) and North East Asia (7%) are most commonly represented [34]. SWSLHD and SLHD are socioeconomically diverse, with contrasting extreme disadvantage and advantage present across the Sydney metropolitan area [35].

### Risk factors

Study variables include: maternal age (categorised as *less than 20*, *20 to 34*, or *over 35 years*); area-based socioeconomic status (SES, *low*, *medium* or *high*); CALD (*yes* or *no*); partner support (*yes* or *no*); maternal history of childhood abuse (*yes* or *no*); history of psychological IPV (*yes* or *no*); history of physical intimate partner violence (IPV, *yes* or *no*); pregnancy known to community services (*yes* or *no*); previous child in out-of-home care (OOHC, *yes* or *no*); alcohol use in pregnancy (*yes* or *no*); history of antenatal health problems (such as gestational diabetes or hypertension, *yes* or *no*); and type of delivery (*normal vaginal*, *assisted vaginal* or *caesarean delivery*)*.*

Area-based SES was measured using the Socio-Economic Index for Areas (SEIFA), and based on the mother’s address. SEIFA is a composite indicator developed by the Australian Bureau of Statistics that ranks areas in Australia according to relative socioeconomic advantage and disadvantage [36]. Deciles of SES were categorised into *high* (top 10% of the population), *medium* (middle 80% of the population) and *low* (bottom 10% of the population), consistent with previous publications [35, 37]. CALD is a term used for communities with diverse characteristics including but not limited to: language, ethnicity, nationality, dress, traditions, food, social structures, art and religion [38]. Data relating to IPV was collected based on the NSW Domestic Violence – Identifying and Responding policy [39], where women are asked: “Within the last 12 months, have you been hit, slapped or hurt in other ways by your partner or ex-partner (physical IPV)?” or “Are you frightened of your partner or ex-partner (psychological IPV)?”

### Outcome variables

The main outcome variables in the present study are antenatal depressive symptoms or distress, and postnatal depressive symptoms or distress, measured using the EPDS. During routine antenatal care, midwives collect psychosocial information including maternal depressive symptoms at the first visit. Non-English speaking pregnant women and mothers may use translated versions of the EPDS where available, which are produced by the New South Wales Multicultural Health Communication Service [40]. Alternatively, women completed the English version of the EPDS through accredited interpreters. Numerous validation studies, using the English version of the EPDS, have demonstrated that a score of 13 or more strikes the optimal balance of sensitivity and specificity in classifying English-speaking women who are experiencing a major depressive episode postnatally [25, 41, 42]. Though this cut-off correctly detects clinical caseness, lower scores of 10–12 are likely to demonstrate the presence of distress, the consequences of which are poorly researched and understood. Research suggests that the EPDS multi-dimensionally measures anxiety [43, 44], dysphoria and depression [45]. The final score is calculated by combining overall scores to achieve a total score out of 30, then coded as a categorical variable (score of *less than 10*, score of *10 to 12*, score of *13 and above*), with a score of 10 to 12 indicating distress and a score of 13 or more suggestive of antenatal depressive symptoms [25].

Data on postnatal depressive symptoms were collected within the first 6 weeks of birth. The total number of postnatal depressive symptoms was also combined to obtain a total score (out of 30) and coded as a categorical variable (score of *less than 10*, score of *10 to 12*, score of *13 and above*). We disaggregated the antenatal and postnatal score (to include 10 to 12) to examine the associations for women who are distressed, but who are not currently offered structured clinical assessment, as the cut-off for assessment remains 13 or more. In the present analysis, EPDS cut-points used to indicate probable depression were selected based on previously published studies [35, 46–48] and the current Australian endorsed guidelines on improving mental health outcomes for parents and infants [49].

The EPDS is the most commonmly used and widely-accepted screening tool for identifying depressive symptoms in the perinatal period worldwide, with a reported sensitivity of 68–86% and specificity of 78–96% [25, 42]. An Australian validation study of 4148 women, reported a sensitivity of 100% and specificity of 89% [41]. An EPDS score of 13 or more has been demonstrated to have a positive predictive value (PPV) for detecting major depression of 70% in the Australian setting [41]. The EPDS has been validated across a range of cultures [41, 42, 50–53] and has demonstrated its superiority to unstructured health professional assessment or routine care both internationally and in Australia [54–56]. The EPDS has been translated into a number of languages, including a validation study conducted in South Western Sydney in Vietnamese and Arabic-speaking women [57]. Women from over 25 countries are represented in this study cohort [37].

### Statistical analysis

The initial analysis involved the calculation of frequencies and prevalence of perinatal distress and depressive symptoms by study factors. Univariate regression models were used to investigate the association between study factors and the outcome measures of perinatal distress and depressive symptoms, followed by multivariate regression models that adjusted for confounders. Predictors of antenatal and postnatal distress or depressive symptoms were investigated in logistic regression models to determine if there was an association with key study factors. The confounding factors considered in the present study were based on previously published reports [20, 58, 59] include alcohol consumption during pregnancy, smoking during pregnancy, sex of the infant, maternal body mass index and birthing facility. A similar analytical strategy was used to account for the potential confounding relationship between study factors and perinatal distress or depressive symptoms. Odds ratios, and their corresponding confidence intervals, were estimated and reported as the measure of association between study factors and outcome variables.

### Missing data

The study also investigated the potential effect of missing data on estimated odds ratios in sensitivity analyses, which were conducted on an imputed data set based on the original cohort comprising complete study factors and outcome data. Multivariate imputation by chained equation was used in the analysis [60], which assumes that data are missing at random and that the characteristics of known participants can be used to estimate the characteristics of participants with missing data [61]. Sensitivity analyses were conducted using the *ice* command in Stata (Stata Corp, V.15.0, College Station, TX, USA), based on 25 multiple imputations [62]. We estimated and reported the revised odds ratios and the corresponding confidence intervals from the imputed dataset, using the *mim* command.

## Results

### Antenatal distress (EPDS 10–12)

The prevalence of antenatal distress was 8.0% in this cohort. This figure was higher in mothers who reported physical intimate partner violence (IPV) (14.1%) and lack of partner support (15.3%). Being from a CALD background (AOR = 2.0, 95% CI 1.79–2.26, *P* < 0.001), maternal experience of childhood abuse (AOR = 1.9, 95% CI 1.61–2.32, *P* < 0.001), having a previous child in out-of-home care (AOR = 1.5, 95% CI 1.19–1.93, *P* < 0.001), having an unsupportive partner (AOR = 2.9, 95% CI 2.30–3.67, *P* < 0.001), reporting alcohol use in pregnancy (AOR = 1.6, 95% CI 1.11–2.32, *P* = 0.011), experience of antenatal medical problems (AOR = 1.2, 95% CI 1.08–1.43, *P* = 0.002) and report of physical IPV (AOR = 1.6, 95% CI 1.10–2.33, *P* = 0.013) were associated with experiencing antenatal distress (defined as EPDS 10–12) compared with women who did not report these factors to be present. Maternal age of < 20 years (AOR = 1.7, 95% CI 1.01–2.85, *P* = 0.045) was associated with antenatal distress compared with women aged between 21 and 34. Pregnant women from higher, compared with lower, socio-economic backgrounds were less likely to experience antenatal distress (AOR = 0.7, 95% CI 0.61–0.91, *P* = 0.004). In this cohort, antenatal distress was not associated with maternal age ≥ 40, “medium” socioeconomic status, report of psychological IPV or if a previous pregnancy was known to child protective services (see Table 1 for figures).

**Table 1:** Study factors and antenatal distress (EPDS = 10–12) in South Western Sydney and Sydney Local Health Districts, 2014–2016 (*N* = 53, 032)

				Complete cases				Multiple imputation*			
Outcome	Participants	Cases	%	Unadjusted OR (95%CI)	*P* value	Adjusted OR (95%CI)	*P* value	Unadjusted OR (95%CI)	*P* value	Adjusted OR (95%CI) (a)	*P* value
Antenatal distress	42,027	3378	8.0								
Maternal age group											
20–34 years	39,199	3119	8.0	1.0		1.0		1.0		1.0	
> 35 years	2184	179	8.2	1.0 (0.9–1.2)	0.60	1.0 (0.8–1.2)	0.768	1.0 (0.9–1.2)	0.550	1.0 (0.8–1.3)	< 0.001
< 20 years	643	79	12.3	1.7 (1.4–2.2)	< 0.001	1.7 (1.0–2.8)	0.045	1.7 (1.4–2.2)	< 0.001	1.7 (1.0–2.8)	< 0.001
SES category											
Low	16,529	1566	9.5	1.0		1.0		1.0		1.0	
Medium	19,345	1374	7.1	0.7 (0.7–0.8)	< 0.001	0.9 (0.8–1.0)	0.066	0.7 (0.7–0.8)	< 0.001	0.9 (0.8–1.0)	< 0.001
High	4701	260	5.5	0.5 (0.5–0.6)	< 0.001	0.7 (0.6–0.9)	0.004	0.5 (0.5–0.6)	< 0.001	0.7 (0.6–0.9)	< 0.001
CALD group											
No	20,668	1245	6.0	1.0		1.0		1.0		1.0	
Yes	20,560	2078	10.1	1.8 (1.7–2.0)	< 0.001	2.0 (1.8–2.3)	< 0.001	1.8 (1.7–2.0)	< 0.001	2.0 (1.8–2.2)	< 0.001
Supportive partner											
Yes	39,735	3096	7.8	1.0		1.0		1.0		1.0	
No	1193	182	15.3	3.0 (2.6–3.6)	< 0.001	2.9 (2.3–3.7)	< 0.001	3.0 (2.5–3.5)	< 0.001	2.9 (2.3–3.6)	< 0.001
Maternal history of childhood abuse											
No	38,020	2903	7.6	1.0		1.0		1.0		1.0	
Yes	2803	342	12.2	1.9 (1.7–2.1)	< 0.001	1.9 (1.6–2.3)	< 0.001	1.9 (1.7–2.1)	< 0.001	1.9 (1.6–2.3)	< 0.001
Psychological IPV											
No	40,367	3209	7.9	1.0		1.0		1.0		1.0	
Yes	560	71	12.7	2.2 (1.7–2.8)	< 0.001	1.2 (0.8–1.9)	0.312	2.2 (1.7–2.8)	< 0.001	1.3 (0.9–2.0)	0.166
Physical IPV											
No	40,529	99	0.2	1.0		1.0		1.0		1.0	
Yes	700	142	14.1	2.4 (1.9–2.9)	< 0.001	1.6 (1.1–2.3)	0.013	2.4 (1.9–2.9)	< 0.001	1.5 (1.0–2.1)	0.035
Pregnancy known to FaCS											
No	35,482	2767	7.8	1.0		1.0		1.0		1.0	
Yes	748	99	13.2	2.0 (1.6–2.5)	< 0.001	1.3 (0.9–1.7)	0.136	2.0 (1.6–2.5)	< 0.001	1.2 (0.9–1.7)	< 0.001
Previous child in OOHC											
No	26,421	1957	7.4	1.0		1.0		1.0		1.0	
Yes	1015	137	13.5	2.1 (1.7–2.5)	< 0.001	1.5 (1.2–1.9)	0.001	2.1 (1.7–2.5)	< 0.001	1.5 (1.2–1.9)	< 0.001
Alcohol use in pregnancy											
No	40,688	3238	7.9	1.0		1.0		1.0		1.0	
Yes	647	78	12.1	1.6 (1.3–2.1)	< 0.001	1.6 (1.1–2.3)	0.011	1.6 (1.3–2.1)	< 0.001	1.5 (1.1–2.2)	0.018
Antenatal health problems											
No	34,322	2641	7.7	1.0		1.0		1.0		1.0	
Yes	6442	611	9.5	1.3 (1.2–1.4)	< 0.001	1.2 (1.1–1.4)	0.002	1.3 (1.2–1.4)	< 0.001	1.3 (1.1–1.5)	< 0.001

In this cohort, an antenatal EPDS score of 10 to 12 was significantly associated with higher odds of having either persistent distress during postnatal follow-up (EPDS 10–12, AOR = 4.3, 95% CI 3.51–5.22, *P* < 0.001) or development of probable postnatal depression (EPDS≥13, AOR = 4.5, 95% CI 3.4–5.9, *P* < 0.001).

### Antenatal depressive symptoms (or high depressive symptoms, EPDS ≥13)

The prevalence of antenatal depressive symptoms was 5.9% in this cohort, but much higher if there was reported presence of psychological or physical IPV (25.0% or 20.3%, respectively) or experience of an unsupportive partner (28.3%). As with antenatal distress, antenatal depressive symptoms were associated with being from a CALD background, maternal experience of child abuse and report of psychological or physical IPV (see Table 2 for figures). Compared with mothers from low socioeconomic backgrounds, women from medium or high socioeconomic backgrounds were less likely to experience antenatal depressive symptoms. Antenatal depressive symptoms were not associated with maternal age group, previous child being known to child protective services or in an out-of-home care placement, alcohol use or medical problems in pregnancy.

**Table 2:** Study factors and antenatal depressive symptoms (EPDS≥13) in South Western Sydney and Sydney Local Health Districts, 2014–2016 (*N* = 53, 032)

				Complete cases				Multiple imputation*			
Outcome	Participants	Cases	%	Unadjusted OR (95%CI)	*P* value	Adjusted OR (95%CI)	*P* value	Unadjusted OR (95%CI)	*P* value	Adjusted OR (95%CI) (a)	*P* value
Antenatal depressive symptoms	42,027	2496	5.9								
Maternal age group											
20–34 years	39,199	2273	5.8	1.0		1.0		1.0			
> 35 years	2184	156	7.1	1.3 (1.1–1.5)	0.008	1.2 (1.0–1.5)	0.101	1.3 (1.1–1.5)	0.008	1.2 (1.0–1.5)	0.123
< 20 years	643	67	10.4	2.0 (1.5–2.6)	< 0.001	0.5 (0.2–1.2)	0.123	2.0 (1.5–2.6)	< 0.001	0.7 (0.3–1.5)	0.320
SES category											
Low	16,529	1272	7.7	1.0	< 0.001	1.0		1.0		1.0	
Medium	19,345	980	5.1	0.6 (0.6–0.7)	< 0.001	0.8 (0.7–0.9)	< 0.001	0.6 (0.6–0.7)	< 0.001	0.8 (0.7–0.9)	< 0.001
High	4701	155	3.3	0.4 (0.3–0.5)	< 0.001	0.6 (0.4–0.7)	< 0.001	0.4 (0.3–0.5)	< 0.001	0.6 (0.4–0.7)	< 0.001
CALD group											
No	20,668	946	4.6	1.0		1.0		1.0		1.0	
Yes	20,560	1510	7.3	1.7 (1.6–1.9)	< 0.001	2.0 (1.8–2.3)	< 0.001	1.7 (1.6–1.9)	< 0.001	2.0 (1.8–2.3)	< 0.001
Supportive partner											
Yes	39,735	2037	5.1	1.0	< 0.001	1.0		1.0		1.0	
No	1193	337	28.3	8.5 (7.4–9.8)	< 0.001	6.5 (5.3–8.0)	< 0.001	8.1 (7.0—9.2)	< 0.001	6.2 (5.0–7.6)	< 0.001
Maternal history of childhood abuse											
No	38,020	1969	5.2	1.0		1.0		1.0		1.0	
Yes	2803	408	14.6	3.3 (3.0–3.8)	< 0.001	3.2 (2.6–3.8)	< 0.001	3.3 (3.0–3-3.8)	< 0.001	3.1 (2.6–3.8)	< 0.001
Psychological IPV											
No	40,367	2269	5.6	1.0		1.0		1.0		1.0	
Yes	560	137	24.5	6.0 (4.9–7.3)	< 0.001	2.6 (1.8–3.7)	< 0.001	6.0 (4.9–7.3)	< 0.001	2.7 (1.9–3.9)	< 0.001
Physical IPV											
No	40,529	2290	5.7	1.0		1.0		1.0		1.0	
Yes	700	142	20.3	4.7 (3.9–5.7)	< 0.001	1.3 (0.9–1.9)	0.150	4.7 (3.9–5.7)	< 0.001	1.4 (0.9–1.9)	0.097
Pregnancy known to FaCS											
No	35,482	2009	5.7	1.0		1.0		1.0		1.0	
Yes	748	108	14.4	3.1 (2.5–3.8)	< 0.001	1.3 (0.9–1.8)	0.141	3.1 (2.5–3.8)	< 0.001	1.3 (0.9–1.8)	0.133
Previous child in OOHC											
No	26,421	1462	5.5	1.0		1.0		1.0		1.0	
Yes	1015	114	11.2	2.3 (1.9–2.9)	< 0.001	1.1 (0.9–1.5)	0.378	2.3 (1.9–2.9)	< 0.001	1.2 (0.9–1.6)	0.187
Alcohol use in pregnancy											
No	40,688	2399	5.9	1.0		1.0		1.0		1.0	
Yes	647	48	7.4	1.3 (1.0–1.8)	0.051	1.0 (0.6–1.7)	0.947	1.3 (1.0–1.8)	0.051	1.2 (0.7–1.8)	0.503
Antenatal health problems											
No	34,322	1937	5.6	1.0		1.0		1.0		1.0	
Yes	6442	451	7.0	1.3 (1.2–1.4)	< 0.001	1.2 (1.0–1.4)	0.073	1.3 (1.2–1.4)	< 0.001	1.2 (1.0–1.4)	0.057

For women with probable antenatal depression (EPDS≥13), the odds of persistence into the postnatal period was significantly raised (AOR = 13.0, 95% CI 10.32–16.47, *P* < 0.001).

### Postnatal distress (EPDS 10–12)

The prevalence of postnatal distress was 5.3% in this cohort. Postnatal distress was associated with being from a CALD background (AOR = 1.3, 95% CI 1.13–1.56, *P* = 0.001), a reported maternal history of child abuse (AOR = 1.4, 1.07–1.80, *P* = 0.013), and having had an assisted vaginal birth, as compared with non-assisted vaginal delivery (AOR 1.6, 95% CI 1.26–2.08, *P* < 0.001). Having a previous child in out-of-home care decreased the likelihood of developing postnatal distress (AOR = 0.6, 95% CI 0.37–0.97, *P* = 0.039), as did being from a higher socioeconomic background (AOR = 1.4, 95% CI 1.07–1.78). Postnatal distress was not associated with maternal age group, having had the pregnancy known to child protective services, lack of partner support, alcohol use or medical problems during pregnancy and finally, reported psychological or physical IPV (see Table 3 for figures).

**Table 3:** Study factors and postnatal distress (EPDS = 10–12) in South Western Sydney and Sydney Local Health Districts, 2014–2016 (*N* = 53, 032)

				Complete cases				Multiple imputation*			
Outcome	Participants	Cases	%	Unadjusted OR (95%CI)	*P* value	Adjusted OR (95%CI)	*P* value	Unadjusted OR (95%CI)	*P* value	Adjusted OR (95%CI) (a)	*P* value
Postnatal distress	40,964	2177	5.3								
Antenatal EPDS											
EPDS 10–12	3378	3378	8.0	3.7 (3.2–4.2)	< 0.001	4.3 (3.5–5.2)	< 0.001	2.9 (2.5–2.3)	< 0.001	3.15 (2.6–3.1)	< 0.001
EPDS ≥13	2496	2496	5.9	4.3 (3.6–5.2)	< 0.001	4.5 (3.4–5.9)	< 0.001	4.0 (3.31–4.3)	< 0.001	4.2 (3.44–5.14)	< 0.001
Maternal age group											
20–34 years	38,064	1994	5.2	1.0		1.0		1.0		1.0	
> 35 years	2265	148	6.5	1.3 (1.1–1.5)	0.005	1.3 (1.0–1.7)	0.057	1.2 (1.0–1.5)	0.015	1.2 (0.9–1.6)	0.116
< 20 years	634	35	5.5	1.1 (0.7–1.5)	0.778	0.6 (0.2–2.0)	0.435	1.1 (0.7–1.5)	0.784	0.6 (0.2–1.8)	0.380
SES category											
Low	15,329	820	5.4	1.0		1.0		1.0		1.0	
Medium	19,597	1022	5.2	1.0 (0.9–1.1)	0.943	1.1 (1.0–1.3)	0.175	1.0 (1.0–1.1)	0.729	1.1 (0.9–1.3)	0.222
High	4844	260	5.4	1.0 (0.9–1.1)	0.448	1.4 (1.1–1.8)	0.013	1.0 (0.9–1.2)	0.795	1.3 (1.0–1.7)	0.033
CALD group											
No	20,898	944	4.5	1.0		1.0		1.0		1.0	
Yes	19,342	1194	6.2	1.4 (1.3–1.5)	< 0.001	1.3 (1.1–1.6)	0.001	1.3 (0.2–1.4)	< 0.001	1.2 (1.1–1.4)	0.007
Supportive partner											
Yes	33,558	1715	5.1	1.0		1.0		1.0		1.0	
No	952	72	7.6	1.63 (1.3–2.1)	< 0.001	0.8 (0.5–1.2)	0.322	1.6 (1.3–2.1)	< 0.001	1.0 (0.6–1.4)	0.838
Maternal history of childhood abuse											
No	31,832	1573	4.9	1.0		1.0					
Yes	2400	183	7.6	1.7 (1.4–1.9)	< 0.001	1.4 (1.1–1.8)	0.013	1.5 (1.3–1.8)	< 0.001	1.3 (1.0–1.6)	0.054
Psychological IPV											
No	33,896	1741	5.1	1.0		1.0		1.0		1.0	
Yes	463	29	6.3	1.3 (0.9–2.0)	0.134	1.0 (0.5–2.0)	0.913	1.3 (0.9–1.9)	0.197	0.8 (0.5–1.6)	0.583
Physical IPV											
No	34,055	1735	5.1	1.0		1.0		1.0		1.0	
Yes	568	45	7.9	1.7 (1.2–2.3)	< 0.001	1.2 (0.6–2.1)	0.624	1.6 (1.2–2.1)	0.003	1.3 (0.8–2.2)	0.342
Pregnancy known to FaCS											
No	30,007	1501	5.0	1.0		1.0		1.0		1.0	
Yes	544	32	5.9	1.3 (0.9–1.8)	0.211	1.5 (0.9–2.4)	0.121	1.2 (0.9–1.7)	0.286	1.4 (0.9–2.2)	0.186
Previous child in OOHC											
No	21,787	1023	4.7	1.0		1.0		1.0		1.0	
Yes	750	32	4.3	0.9 (0.6–1.3)	0.644	0.6 (0.4–1.0)	0.039	1.0 (0.7–1.4)	0.920	0.8 (0.5–1.2)	0.220
Alcohol use in pregnancy											
No	35,181	1832	5.2	1.0		1.0		1.0		1.0	
Yes	545	28	5.1	1.0 (0.7–1.4)	0.930	0.9 (0.5–1.7)	0.681	1.0 (0.7–1.5)	0.986	1.1 (0.6–1.8)	0.814
Antenatal health problems											
No	34,167	1802	5.3	1.0		1.0		1.0		1.0	
Yes	5783	322	5.6	1.1 (0.9–1.2)	0.293	1.0 (0.8–1.2)	0.907	1.0 (0.9–1.2)	0.433	1.0 (0.9–1.0)	0.116
Type of delivery											
Normal vaginal	24,921	1157	4.6	1.0		1.0		1.0		1.0	
Assisted vaginal	4649	316	6.8	1.5 (1.3–1.7)	< 0.001	1.6 (1.3–2.1)	< 0.001	1.4 (1.2–1.6)	< 0.001	1.5 (1.2–1.9)	< 0.001
Caesarean section	11,299	695	6.2	1.4 (1.2–1.5)	< 0.001	1.1 (0.9–1.3)	0.360	1.3 (1.2–1.4)	0.024	1.1 (0.9–1.3)	0.435

### Postnatal depressive symptoms (or high depressive symptoms, EPDS ≥13)

The prevalence of postnatal depressive symptoms was 3.1% in this cohort, but much higher if there was reported presence of psychological IPV (10%). As with postnatal distress, postnatal depressive symptoms were associated with being from a CALD background, maternal experience of child abuse, report of psychological or physical IPV and having an assisted vaginal delivery (see Table 4 for figures). Additionally, postnatal depressive symptoms were significantly associated with having had the pregnancy known to child protective services, lack of partner support and having had a Caesarean birth.

**Table 4:** Study factors and postnatal depressive symptoms (EPDS≥13) in South Western Sydney and Sydney Local Health Districts, 2014–2016 (*N* = 53, 032)

				Complete cases				Multiple imputation*			
Outcome	Participants	Cases	%	Unadjusted OR (95%CI)	*P* value	Adjusted OR (95%CI)	*P* value	Unadjusted OR (95%CI)	*P* value	Adjusted OR (95%CI) (a)	*P* value
Postnatal depressive symptoms	40,964	2177	3.1								
Antenatal EPDS											
EPDS 10–12	3378	3378	8.0	4.3 (3.2–4.2)	< 0.001	4.5 (3.4–5.9)	< 0.001	3.2 (2.7–3.8)	< 0.001	3.1 (2.4–4.1)	< 0.001
EPDS ≥13	2496	2496	5.9	12.0 (10.3–13.9)	< 0.001	13.0 (10.3–16.5)	< 0.001	8.2 (7.0–9.5)	< 0.001	7.9 (6.3–9.9)	< 0.001
Maternal age group											
20–34 years	38,064	1171	3.1	1.0		1.0		1.0		1.0	
> 35 years	2265	99	4.4	1.5 (1.2–1.8)	< 0.001	1.4 (1.0–2.0)	0.059	1.3 (1.1–1.7)	0.004	1.3 (0.9–1.8)	0.169
< 20 years	634	16	2.5	0.8 (0.5–1.3)	< 0.001	1.0 (0.3–3.2)	0.954	0.9 (0.6–1.4)	0.658	0.8 (0.2–2.5)	0.684
SES category											
Low	15,329	563	3.7	1.0		1.0		1.0		1.0	
Medium	19,597	546	2.8	0.8 (0.7–0.8)	< 0.001	1.0 (0.8–1.2)	0.632	0.8 (0.7–0.9)	< 0.001	1.0 (0.8–1.2)	0.661
High	4844	139	2.9	0.8 (0.6–0.9)	0.008	0.9 (0.6–1.3)	0.495	0.8 (0.7–1.0)	0.035	1.0 (0.7–1.4)	0.895
CALD group											
No	20,898	552	2.6	1.0		1.0		1.0		1.0	
Yes	19,342	723	3.7	1.5 (1.3–1.6)	< 0.001	1.6 (1.3–2.0)	< 0.001	1.4 (1.2–1.5)	< 0.001	1.4 (1.1–1.7)	0.002
Supportive partner											
Yes	33,558	946	5.1	1.0		1.0		1.0		1.0	
No	952	86	7.6	3.5 (2.8–4.5)	< 0.001	1.6 (1.1–2.3)	0.017	3.5 (2.8–4.4)	< 0.001	2.0 (1.4–2.8)	< 0.001
Maternal history of childhood abuse											
No	31,832	867	2.7	1.0		1.0		1.0		1.0	
Yes	2400	147	6.1	2.4 (2.0–2.9)	< 0.001	1.9 (1.4–2.5)	< 0.001	2.1 (1.8–2.5)	< 0.001	1.6 (1.2–2.1)	< 0.001
Psychological IPV											
No	33,896	990	2.9	1.0		1.0		1.0		1.0	
Yes	463	46	9.9	3.7 (2.7–5.1)	< 0.001	1.8 (1.0–3.2)	0.062	3.1 (2.3–4.3)	< 0.001	1.45 (0.8–2.5)	0.180
Physical IPV											
No	34,055	1000	2.9	1.0		1.0		1.0		1.0	
Yes	568	46	8.1	3.0 (2.2–4.1)	< 0.001	1.1 (0.6–2.1)	0.667	2.6 (1.9–3.5)	< 0.001	1.0 (0.6–1.8)	0.925
Pregnancy known to FaCS											
No	30,007	849	2.8	1.0		1.0		1.0		1.0	
Yes	544	44	8.1	3.1 (2.2–4.2)	< 0.001	1.9 (1.2–3.1)	0.009	2.5 (1.8–3.4)	< 0.001	1.6 (0.1–2.5)	0.053
Previous child in OOHC											
No	21,787	588	2.7	1.0		1.0		1.0		1.0	
Yes	750	31	4.1	1.5 (1.1–2.2)	0.020	0.7 (0.5–1.2)	0.242	1.5 (1.0–2.1)	0.043	0.8 (0.5–1.3)	0.437
Alcohol use in pregnancy											
No	35,181	1065	3.0	1.0		1.0		1.0		1.0	
Yes	545	15	2.8	0.9 (0.5–1.5)	0.708	0.7 (0.3–1.8)	0.503	1.0 (0.6–1.6)	0.865	0.8 (0.3–1.8)	0.587
Antenatal health problems											
No	34,167	1024	5.3	1.0		1.0		1.0		1.0	
Yes	5783	216	3.7	1.3 (1.1–1.5)	0.002	0.9 (0.7–1.2)	0.588	1.2 (1.0–1.4)	0.013	1.0 (0.9–1.0)	0.094
Type of delivery											
Normal vaginal	24,921	684	2.7	1.0		1.0		1.0		1.0	
Assisted vaginal	4649	161	3.5	1.3 (1.1–1.6)	0.003	1.6 (1.1–2.2)	0.014	1.2 (1.0–1.5)	0.024	1.4 (1.0–1.9)	0.070
Caesarean section	11,299	440	3.9	1.5 (1.3–1.7)	< 0.001	1.4 (1.1–1.7)	0.002	1.3 (1.2–1.5)	< 0.001	1.3 (1.1–1.6)	0.009

*OOHC* Out-of-home care, *FaCS* Family and Community Services, *IPV* Intimate partner violence

Sensitivity analysis of the revised odds ratios and corresponding confidence intervals were not significantly different from the complete imputed case analysis, indicating that missing data did not substantially affect the observed findings.

## Discussion

The prevalence of antenatal distress in this cohort was estimated at 8.0% and shares higher odds of coming from a CALD or low SES background, a maternal history of experiencing child abuse and lack of partner support, with probable antenatal depression (prevalence of 5.9%). The prevalence of postnatal distress is estimated at 5.3% and shares risk factors with women whose scores indicate probable postnatal depression (prevalence of 3.1%), including being from a CALD background, a maternal history of experiencing child abuse and having had an assisted vaginal delivery.

In this cohort, pregnant women experiencing distress during their pregnancy have four times higher odds of experiencing ongoing distress postnatally or deteriorating further and developing probable postnatal depression, compared with women who scored 9 or less on EDPS screening during their pregnancy. To our knowledge, this is the first time a longitudinal quantitative association has been reported. Currently women signalling distress (EPDS score 10–12) may be offered reassessment within 2 to 4 weeks [49]. If this reassessment reveals ongoing distress with a sub-threshold score, specific interventions are not necessarily offered. If reapplication demonstrates score creep to 13 or more, mental health assessment is usually offered. A criticism of this approach is that the response becomes manualised and crisis driven and is more likely to require intensive, costly treatment and cause potentially preventable suffering. This data supports treating women who score 10 to 12 more proactively and viewing these scores as existing on a continuum where increasing symptomatology produces a higher score, rather than presence of illness being viewed in a binary format, as the current scoring system reflects, and therefore how the data collected were chosen to be analysed.

In 2008, the Australian Government provided $85 million to fund the National Perinatal Depression Initiative [26]. These funds were allocated to state and territory governments with the purpose of delivering universal screening in the antenatal and postnatal period and respond to women who were either experiencing depression or at risk of developing depression. *Beyondblue*, an independent non-profit mental health organisation, was tasked with supporting implementation of this initiative. At the conclusion of this 5 year roll-out, *beyondblue* identified national analytics and integration with support services as areas of program development that were not yet systematic in their delivery [63]. In recent years, New South Wales Health has adopted an integrated care strategy with the primary objective of delivering cohesive care to targeted patient groups [64]. Underpinned by a proportionate universalism and health equity philosophy, this approach scales and intensifies intervention in response to identified need [65]. This strategy could inform an integrated response to risks identified during routine psychosocial assessment and exploit the stratification characteristics of the EPDS.

This study has a number of policy implications. The work corroborates previous research in identifying a range of risk factors for antenatal and postnatal depressive symptoms [17, 20, 66]. It adds to the growing body of literature suggesting that specific attention should be paid to women who are distressed though not necessarily clinically depressed during pregnancy, demonstrating a number of shared risk factors with women who develop probable depression [22, 67]. Using the properties of the EPDS in this way emphasises managing symptoms and function, and shifts focus away from only intervening when there is a definable psychiatric diagnosis. Current practice may dismiss pregnant women’s distress as it is considered ‘expected’ or because it is predominantly characterised by anxiety. A novel finding of this study is the demonstration that antenatal EPDS scores can be used to predict the likelihood of postnatal psychological distress or depressive symptoms. We have shown that women scoring 10 to 12 on the EPDS, who are likely to be distressed, are currently underserved by policy recommendations for repeat testing, rather than offering intervention with a preventative focus.

Policy recommendations for appropriate intervention could draw from the breadth of experimental-control trials that have applied non-pharmacological interventions with the aim of preventing postpartum depression. Evidence of efficacy is varied, though interventions exert greater effect if a risk stratification procedure is applied as a condition of eligibility [68]. These interventions include psychological debriefing post birth [69–73], interpersonal, couples-based and cognitive-behavioural therapies [74–82], antenatal and postnatal classes [83–89], professional or lay-based home visiting [90–94], lay-based telephone support [95], continuity of care and carer [96–98], early and flexible postpartum care [99], web- and computer-based programs [100–103], and mindfulness-based therapies [104].

### Study limitations and strengths

Limitations in study design have been considered by the authors. Firstly, a source of recall and measurement bias is introduced by the self-report nature of a range of questions asked of women during antenatal and postnatal psychosocial assessment. This bias may be introduced in either direction, causing over- or underestimation of the association between risk factor and depressive symptoms. Secondly, this analysis relies on routinely collected and human-scored service data which may present a range of limitations including inaccurate input, coding errors, meaningfully different missing data and an inability to establish temporal correlation between variables and antenatal depressive symptoms collected contemporaneously. The dataset is linked to postnatal data however, which means a temporal relationship between variables and postnatal depression can be established. Thirdly, there are a number of important study factors, such as level of social support outside an intimate partner relationship, past history of psychiatric illness, type of antenatal health problem and markers of pregnancy and parenting preparedness that are not reflected in this dataset and may represent important mediating relations. Fourth, this dataset does not provide an indication of whether women experiencing distress or depression are presenting with these symptoms for the first time, which has implications for risk stratification and management. Fifth, distress is a generic term that fails to capture a range of contributory causes such as trauma or stressor-related conditions. Sixth, we acknowledge that women with long-standing mental illness may not score highly on EPDS screening, either because of the chronicity of their illness or concern regarding the consequences of a high-score. Seventh, the lack of a specific assessment for the outcome variables among women from various cultural backgrounds is another limitation of the study given the varied EPDS cut-offs used in examining probable perinatal depression in these populations. Specific evidence pertaining to women from CALD communities signalling antenatal or postnatal distress and/or depressive symptoms may be helpful in targeted advocacy and policy interventions. We recognise that in current service delivery the cut-off score derived from English-speaking population studies is applied to all women, regardless of whether the EPDS was completed in its translated form. Furthermore, it is not well understood to what extent translated versions of the EPDS, or translators, are used in these settings. Lastly, this dataset covers the majority of women across the two health districts, but misses those who have their antenatal and delivery care privately. This could introduce a degree of reporting bias as the distribution of maternal age, socioeconomic status and other risk factors may be different between cohorts.

Despite these limitations, there are a range of study strengths. Firstly, we take advantage of routinely collected data that reflects a diverse cohort of women and can be used to inform contextually appropriate policy and practice. As Australian healthcare systems have moved to electronic medical records, the Australian Government released principles of data integration, calling for the strategic use of available resources to drive research and policy justification [105]. Three advantages to the approach taken for this study include (1) the cost-effectiveness of analysing routinely collected data and ensuring a return on public investment; (2) the use of real-world data to evaluate the impact of delivered policy; and (3) the use of internally linked data to provide a longitudinal picture and allow causal inference.

Another study strength is the application of multiple imputations to decrease the potential for bias secondary to missing data. Odds ratios were calculated with the complete dataset and again with the imputed dataset assuming the data were missing at random. Sensitivity analysis reveals that results were unlikely to be affected by missing data.

The EPDS, as with any screening tool, has had a range of criticisms levelled against aspects of its delivery, use and interpretation, reviewed recently by Matthey and Agostini [106]. We are aware that many women who are experiencing a major depressive episode may not, and should not, be identified by EPDS screening alone and conversely women who score highly may not be experiencing a major depressive episode. We are also aware that there is conjecture in the literature as to whether the EPDS has uni- or multidimensional psychometric properties and the use of a range of cut-off scores in validation studies across a range of cultures and pregnancy time-points [107]. Notwithstanding these criticisms, its wide and routine use in Australia makes it a central tool for research and evidence-informed policy. Furthermore, while the EPDS scores would be viewed as a continuum result, current Australian policy recommendations around perinatal depression screening do not support the continuum approach.

## Conclusion

Women scoring 10 to 12 on the EPDS during both antenatal and postnatal assessment share a similar burden of risk factors when compared with women scoring 13 or more. These include coming from a CALD background, maternal experience of childhood abuse and presence of psychological or physical IPV. Women experiencing distress (scoring 10 to 12) on antenatal assessment are at high risk for persisting distress or developing postnatal depressive symptoms (scoring 13 or more postnatally). This study suggests that while screening for mood disorder in pregnancy is appropriate and necessary, a singular cut-off of 13 or more for further assessment is not supported by this analysis. Risk stratification and integration of population-level preventative interventions is one such instrument that should be further explored.

## Data Availability

Only listed member on the ethics approval are able to access the data used for this analysis. Any data requests or queries need to be forwarded to: South Western Sydney Local Health District Ethics Committee (Research and Ethics Office, Locked Bag 7103, Liverpool BC, NSW, 1871; Phone: + 61 (02) 87388304; Fax: + 61 (02) 87388310; E: research.support@sswahs.nsw.gov.au; and Sydney Local Health District Ethics committee c/− Research Ethics and Governance Office (REGO), Royal Prince Alfred Hospital, Missenden Road CAMPERDOWN, NSW, 2050; Phone: + 61 (02) 95156766; Facsimile: + 61 (02) 95157176.

## Abbreviations

CALD: Culturally and linguistically diverse
EPDS: Edinburgh Postnatal Depression Scale
FaCS: Family and Community Services
IPV: Intimate partner violence
NSW: New South Wales
OOHC: Out-of-home-care
SES: Socio-economic status
SLHD: Sydney Local Health District
SWSLHD: South Western Sydney Local Health District
